# Middle Cerebral Artery M2 Thrombectomy: Safety and Technical Considerations in the German Stroke Registry (GSR)

**DOI:** 10.3390/jcm11154619

**Published:** 2022-08-08

**Authors:** Moriz Herzberg, Franziska Dorn, Christoph Trumm, Lars Kellert, Steffen Tiedt, Katharina Feil, Clemens Küpper, Frank Wollenweber, Thomas Liebig, Hanna Zimmermann

**Affiliations:** 1 Institute of Neuroradiology, Ludwig Maximilian University (LMU), 80539 Munich, Germany; 2 Department of Radiology, University Hospital Würzburg, 97080 Würzburg, Germany; 3 Department of Neuroradiology, University Hospital Bonn, 53127 Bonn, Germany; 4 Department of Neurology, Ludwig Maximilian University (LMU), 80539 Munich, Germany; 5 Institute for Stroke and Dementia Research (ISD), University Hospital, Ludwig Maximilian University (LMU), 80539 Munich, Germany; 6 Department of Neurology, University Hospital Tübingen, 72076 Tübingen, Germany; 7 Department of Neurology, Hospital Wiesbaden, 65189 Wiesbaden, Germany

**Keywords:** mechanical thrombectomy, M2, distal occlusion, endovascular therapy, aspiration, stent retriever, outcome

## Abstract

There is ongoing debate concerning the safety and efficacy of various mechanical thrombectomy (MT) approaches for M2 occlusions. We compared these for MT in M2 versus M1 occlusions. Subgroup analyses of different technical approaches within the M2 MT cohort were also performed. Patients were included from the German Stroke Registry (GSR), a multicenter registry of consecutive MT patients. Primary outcomes were reperfusion success events. Secondary outcomes were early clinical improvement (improvement in NIHSS score > 4) and independent survival at 90 days (mRS 0–2). Out of 3804 patients, 2689 presented with M1 (71%) and 1115 with isolated M2 occlusions (29%). The mean age was 76 (CI 65–82) and 77 (CI 66–83) years, respectively. Except for baseline NIHSS (15 (CI 10–18) vs. 11 (CI 6–16), *p* < 0.001) and ASPECTS (9 (CI 7–10) vs. 9 (CI 8–10, *p* < 0.001), baseline demographics were balanced. Apart from a more frequent use of dedicated small vessel stent retrievers (svSR) in M2 (17.4% vs. 3.0; *p* < 0.001), intraprocedural aspects were balanced. There was no difference in ICH at 24 h (11%; *p* = 1.0), adverse events (14.4% vs. 18.1%; *p* = 0.63), clinical improvement (62.5% vs. 61.4 %; *p* = 0.57), mortality (26.9% vs. 22.9%; *p* = 0.23). In M2 MT, conventional stent retriever (cSR) achieved higher rates of mTICI3 (54.0% vs. 37.7–42.0%; *p* < 0.001), requiring more MT-maneuvers (7, CI 2–8) vs. 2 (CI 2–7)/(CI 2–2); *p* < 0.001) and without impact on efficacy and outcome. Real-life MT in M2 can be performed with equal safety and efficacy as in M1 occlusions. Different recanalization techniques including the use of svSR did not result in significant differences regarding safety, efficacy and outcome.

## 1. Introduction

Mechanical thrombectomy (MT) has been extensively proven for patients with proximal large vessel occlusions (LVO) in the anterior circulation [1]. MT is recommended for all patients with distal internal carotid artery (ICA) and/or M1 occlusion types and supported by class I evidence [2]. However, there is limited available data for patients with distal middle cerebral artery (MCA) occlusions. Current guidelines are inexplicit and do not contain specific endovascular treatment recommendations [2]. Even though M2 branches typically supply less parenchymal volumes, functional independence at 90 days is only achieved in half of untreated patients [3]. Partial or complete recanalization is achieved only in 52% of M2 occlusions by intravenous recombinant tissue plasminogen activator (rTPA) alone [4]. In large randomized multicenter MT trials, patients with M2 occlusions were underrepresented. MR CLEAN was the only randomized trial that explicitly included patients with M2 occlusions [5]. An M2-subgroup meta-analysis by the HERMES Collaborators (n = 130) found a treatment effect in favor of MT [6]. Pooled data (n = 131) from SWIFT, STAR, DEFUSE 2, and IMS III found that successful reperfusion after M2-thrombectomy is associated with excellent functional outcomes (mRS 0–1; OR 2.2. 95% CI 1.0–4.7) [7]. Further data from various single-center studies indicate that MT is safe and technically successful in M2 occlusions [8,9,10]. However, vascular injury may be more common in smaller diameter vessels with conventional stent retrievers, and as a result, the rate of hemorrhagic complications may be higher in M2 rather than in M1 occlusions [9,11,12,13]. Furthermore, not all M2 occlusions are accessible using distal aspiration catheters, potentially making direct contact aspiration or a combined technique such as SAVE (stent retriever assisted vacuum-locked extraction) impossible [14,15].

MT is based on two basic principles: stent retriever assisted manual retrieval of the clot material or contact aspiration using a large diameter distal access catheter, or both.

Stent retrievers are classified into conventional stent retrievers (cSR) or small vessel stent retrievers (svSR) based on their diameter, with a diameter of up to 3.5 mm defining a small stent retriever.

According to manufacturers, svSR have larger cell sizes when compared to cSR, maximizing clot integration when deployed in small vessels. Deployment and retrieval safety have already been optimized for a more distal use, with softer distal tips and decreased radial force when compared with larger stent retrievers. This should minimize the odds of vessel perforation and endothelial damage in smaller vessels [13,16].

When using a stent retriever, the occlusion is passed through using a microcatheter, and the retractable stent is then unsheathed therefrom. After release, the stent retriever integrates into the clot material over a short period of time (usually between two and four minutes). The stent and microcatheter are then removed together with the thrombus.

In contact aspiration a distal access catheter is advanced to the proximal portion of the thrombus using a microcatheter, and after docking the tip to the thrombus, the microcatheter is removed and aspiration is performed with a vacuum syringe over a period of at least two minutes. The catheter is then withdrawn under continuous aspiration [15].

Contrary to MT in large vessel occlusions (LVOs), the best technique for distal occlusions has not yet been determined [11].

We sought to evaluate real-world data from the German Stroke Registry [17] by comparing adverse events, clinical and angiographic outcomes of patients with M1 and M2 occlusions.

## 2. Materials and Methods

### 2.1. Study Population

Patient data were collected as part of the GSR (ClinicalTrials.gov Identifier: NCT03356392) between June 2015 and December 2019. The 25 participating centers reflect both primary and comprehensive stroke centers (12 university hospitals and 13 municipal hospitals). A detailed description of the GSR study design and main outcome data was published elsewhere [17,18].

**Figure 1:**
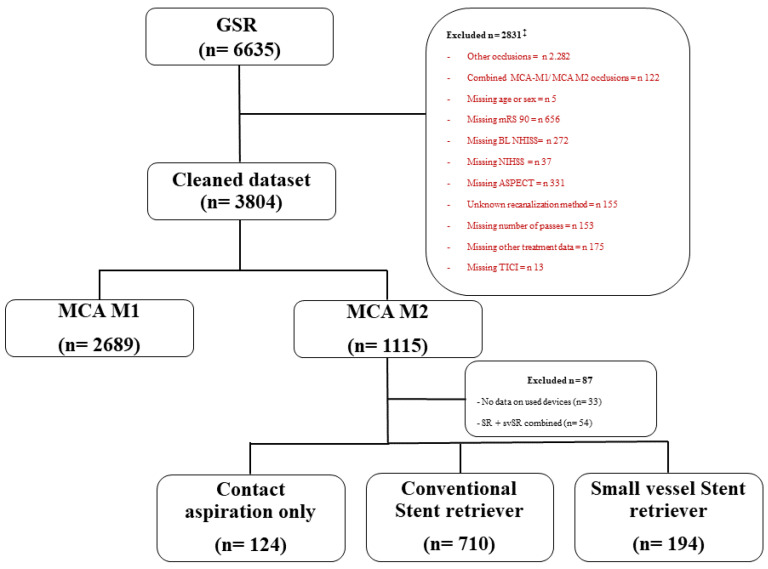
Flowchart of patient selection and exclusion criteria. GSR: German stroke registry; MCA: middle cerebral artery; mRS: modified Rankin Scale; NIHSS: National Institutes of Health Stroke Scale. ‡ multiple missing data within one excluded dataset possible.

We retrospectively included patients from the GSR cohort with angiographically confirmed M1 and M2 occlusions after data clearing for relevant missing values (Figure 1).

Out of 6635 patients in the GSR registry 3804 patients had M1 or M2 occlusions. After exclusion of patients with combined occlusions and missing clinical (for example mRS, NIHSS, ASPECT) or periprocedural data (recanalization method, number of passes), 2689 M1 occlusions and 1115 M2 occlusions were available for analysis.

In a subgroup analysis we further evaluated contact aspiration (CA) alone as compared with conventional stent retriever (cSR) ± CA and small vessel stent retriever (svSR) ± CA.

### 2.2. Definition of Middle Cerebral Artery M2 Segment

The M2 segment was defined as the vertical branches of the MCA in the Sylvian fissure, which originate at the genu and extend to the next genu at the level of the operculum [11,19]. Only primary M2 occlusions were included in our study, while secondary clot migrations were excluded. Patients with combined LVO and M2 occlusions were excluded.

### 2.3. Image Acquisition and Analysis

The detailed imaging protocols for GSR participating sites are described in detail elsewhere [17]. Each site’s imaging protocol included a non-contrast-enhanced computed tomography (NCCT) scan and a CT angiogram (CTA) at baseline. CT Perfusion Imaging (CTP) and Magnetic Resonance Imaging (MRI) with and without perfusion could be acquired additionally. Local neuroradiologists evaluated theASPECTS [20] and modified treatment in cerebral infarction (mTICI) [21] score.

### 2.4. Treatment

All eligible patients received intravenous rTPA prior to MT. The choice of MT approach was made at the discretion of the neuroradiologist, choosing from CA or cSR/svSR, or combinations thereof. The procedure was performed either under conscious sedation (CS) or general anesthesia (GA) according to the local standard operating procedures (SOPs). For patients with ipsilateral ICA stenosis (NASCET > 70%) angioplasty with or without stenting could be performed.

The distal access catheter or the SR was selected at the discretion of the responsible interventionalist depending on the vessel diameter. Due to the multicenter approach, a variety of different catheters and stent retrievers were used.

The small vessel stent retrievers used were: Trevo XP ProVue Retriever 3 × 20 mm (Stryker Neurovascular, Fremont, CA, USA), ERIC 3 × 15 mm und 3 × 20 (MicroVention, Tustin, CA, USA), pRESET LITE 3 × 20 mm (Phenox, Bochum, Germany), Catch mini 3 × 15 mm (Balt; Montmorency, France), Tigertriever 17 0.5–3 × 23 mm; Tigertriever 13 0.5–2.5 × 20.5 mm (Rapid Medical, Yokneam, Israel), Aperio Hybrid 2.5 × 16 mm, 3.5 × 28 mm (Acandis, Pforzheim, Germany).

### 2.5. Outcome and Safety

The primary outcome measures were reperfusion success and adverse events. The secondary outcome measures were functional independence and clinical improvement. Technical recanalization success was defined as mTICI ≥ 2b according to the modified treatment in cerebral infarction (mTICI) score [21]. Adverse events included any intracerebral hemorrhage (ICH) within 24 h after thrombectomy in follow-up imaging, subarachnoid hemorrhage (SAH), and angiographic vasospasm, recurrent stroke at 24 h, hemorrhagic transformation at 24 h and death at 90 days. Good functional outcome, defined as a modified Rankin Scale (mRS) score between 0–2 at 90 days, was assessed by trained neurologists through an interview, either face-to-face or over the telephone. Clinical improvement was defined as an improvement in the National Institutes of Health Stroke Scale (NIHSS) score from admission to discharge of >4.

### 2.6. Statistical Analysis and Outcome Model Description

Standard descriptive statistics were presented for categorical variables (mean, SD, and median with interquartile range were used for continuous variables and frequency distributions).

For between-group comparisons, χ^2^ tests and Fisher’s exact tests were used for categorical variables, whereas *t*-tests and Wilcoxon rank-sum tests were used for continuous variables, when appropriate. Analyses were performed using R (Rstudio, Boston, MA, USA, version 3.6.3). A level of significance of alpha = 0.05 was used.

## 3. Results

### 3.1. Patient Selection

From 6635 patients in the GSR, 3804 patients fulfilled the inclusion criteria (Figure 1). Of these, 87 patients were excluded because of missing data entries on the device used or the combined use of cSR + svSR. Within the M2 cohort, 124 patients were treated with CA only. Within the cohort treated with stent retriever ± aspiration, we defined the subgroups cSR (n = 170) and svSR (n = 194) (Figure 1).

### 3.2. Baseline Characteristics, Clinical Outcome, and Safety M1 vs. M2

**Figure 2:**
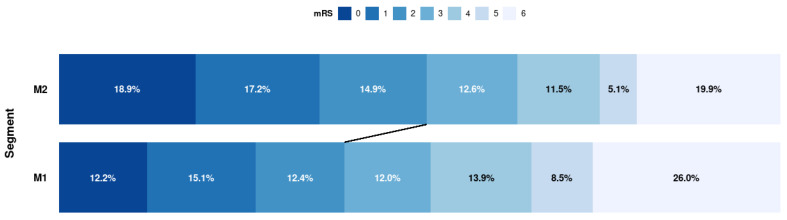
Flowchart of patient selection and exclusion criteria. Distribution of scores on the modified Rankin Scale (mRS) at 90 days. Shown is the shift in outcome within the study population (M1: n = 2689; M2: n = 1115). The numbers represent the percentage (rounded to first digit) of patients for a given outcome and group.

Apart from differences in baseline NIHSS and ASPECTS scores, the baseline characteristics were balanced between the M1 and M2 cohort. Detailed baseline characteristics and treatment details are shown in Table 1. Details on missing individual variables are presented in Tables S1 and S2 (online only supplement). The clinical outcome analysis, procedural results, and safety outcome are shown in Table 2 and Figure 2. A good clinical outcome (mRS 0–2) was achieved significantly more frequently in the M2 group (M1 38.9% vs. M2 46.5%; *p* ≤ 0.001) (Figure 2). Neither improvement of clinical symptoms (62.5% vs. 61.4%; *p* = 0.57), mortality (26.9% vs. 22.9%; *p* = 0.23), nor periprocedural adverse events (14.4% vs. 18.1%; *p* = 0.63), SAH (1.6% vs. 2.6%; *p* = 0.53), vasospasm (3.6% vs. 4.4%; *p* = 0.26), nor recurrent stroke (3.4% vs. 3.9%; *p* = 0.44) differed between the M1 or M2 cohort. There was no increased rate of ICH at 24 h (11.1% vs. 11.1%, *p* = 1.0). M2 occlusions were significantly more often treated with dedicated svSR (3.0% vs. 17.4%; *p* ≤ 0.001). The mean number of passes was identical between cohorts, with the maximum number being higher in the M1 cohort (M1: CI 2–8 vs. M2: CI 2–7; *p* = 0.10). A first pass mTICI 2b/3 was rare in both groups, but more frequently achieved in patients with M2 occlusions (1.3% vs. 2.7%; *p* = 0.50). Treatment was completed in a higher proportion of M1 occlusions (94.2% vs. 91.6%; *p* = 0.40).

### 3.3. Subgroup Analysis of the M2 Cohort Regarding Treatment Strategies

Baseline characteristics were balanced between the different treatment strategies within the M2 cohort (Table 3). There was no significant difference in good functional outcome (mRS 0–2) between CA, cSR and svSR at 90-days (45.7–51.8%; *p* = 0.65), neither in neurological improvement at discharge (60.5–71.4%; *p* = 0.98), mortality (20.7–23.4%; *p* = 0.74) or periprocedural complications (14.5–21.6%; *p* = 0.20) (Table 4). The median number of passes was significantly higher for the cSR group when compared to the CA and svSR groups (7 (CI 2–8) vs. 2 (CI 2–7)/(CI 2–2); *p* ≤ 0.001). While only the CA group achieved a significantly the higher rate of first pass mTICI 2b/3, the overall frequency of first pass success was actually rather low (4.2% vs. 0–0.1%; *p* ≤ 0.001). The cSR group had the highest rate of mTICI 3 (37.7% vs. 42.0 vs.54.0%; *p* ≤ 0.001), however required the highest number of passes (7 vs. 2) and its use did not impact clinical outcome or mortality. Between the different MT technique cohorts, no difference was found regarding risk for SAH (4.1% vs. 0.8% and 2.8%; *p* = 0.22), and induction of angiographic vasospasm (5.5% vs. 3.2% and 2.6%; *p* = 0.17).

## 4. Discussion

Limited data is available on MT of M2 occlusions, in contrast to the case of LVO. The available studies indicate that MT is technically feasible with successful recanalization rates (TICI ≥ 2b) between 69.1% [22] and 86.9% [1]. Our results are also in this range (83%), comparable to those of a recent meta-analysis (81%) [23], and higher than in the Hermes registry (71%) [6]. As far as the available literature shows, the chance for successful recanalization is equal or even higher for M2 than for M1 occlusions [9,14,24,25]. Compared to the STRATIS-Registry, the overall results for good clinical outcome and mortality within the GSR are less favorable, especially when considering M1 occlusions [9]. Keeping this in mind, we found a higher rate of good (mRS 0–2) and excellent functional outcome (mRS 0–1) in the M2 group when compared to the M1 group, different from the M2 group of the STRATIS registry and a subgroup analysis of the ARISE II trial [8,9]. The initially lower baseline NIHSS score and the potentially smaller parenchymal damage [26] might be an explanation for this finding. However, to estimate the true treatment effect, randomized trials including a control group will be necessary. Our results are in keeping with a recent meta-analysis by Saber et al. who found similar reperfusion rates (84.9% versus 84.2%) in M1 occlusions when compared to M2 occlusions, yet with significantly higher rates of functional independence in M2 occlusion patients (OR, 1.46 [95% CI, 1.15–1.86]; *p* = 0.002) [23]. Similarly, a further meta-analysis reported better functional outcomes (48.6% versus 43.5%; OR, 1.24 [95% CI, 1.05–1.47], *p* = 0.01) and lower mortality in patients treated with MT in M2 occlusions when compared with M1 occlusions (16.3% versus 20.7%, OR, 0.73 [95% CI, 0.57–0.94], *p* = 0.01) [27]. There was no difference in mTICI scores between M1 and M2 occlusions. We also believe that a more tailored expanded treatment in cerebral infarction (eTICI) score, and especially the recently proposed ER-PMC (eloquent motor cortex-tissue reperfusion score) [28], will help to better estimate reperfusion success for distal occlusions as only the eloquence of the affected area and the affected territory downstream of the occlusion, instead of the entire middle cerebral artery territory are considered [26]. However, as most studies use the mTICI score, this was used for comparability while accepting a potential inherent bias [29].

Within the GSR, patients undergoing MT for M1 and M2 occlusions had similar rates of periprocedural adverse events and ICH at 24 h on follow-up imaging. M2 occlusions were significantly more often treated using dedicated small vessel stent retrievers (3% vs. 17.4%), while the use of a conventional stent retriever more often resulted in mTICI 3 reperfusion. This however required a higher number of retrieval maneuvers (7 vs. 2) without affecting clinical improvement, good functional outcome, or mortality. 

A clinical outcome of mRS 0–2 was achieved significantly more often in patients with isolated M2 occlusions when compared to those exhibiting M1 occlusions. However, M2 occlusion patients were less severely affected in terms of NIHSS and ASPECTS on admission. While this may seem to relativize treatment success in comparison to MT of M1 occlusions, it has already been shown that successful M2 recanalization is associated with improved outcomes (OR 4.22; 95% CI 1.96 to 9.1) when compared with poor or no M2 recanalization (TICI 0–2a) [22], and it thus should not be an argument against MT in M2 occlusions. Furthermore, we found no difference in rates of periprocedural adverse events (14.4 vs. 18.1), ICH (11%), SAH (1.6% vs. 2.6%), vasospasm (3.6% vs. 4.4%), or recurrent stroke (3.4% vs. 3.9%) between patients with M1 or M2 occlusions. This is in line with most previous studies and a recent meta-analysis [27,30,31], but contradicts findings in the STRATIS registry that found higher rates of ICH in M2 occlusions when compared to M1 occlusion (4% vs. 1%; *p* = 0.01) [9], and inSaber et al. (15% vs. 4.7% ICH) in their meta-analysis [23]. In MR CLEAN, procedural complications (28% vs. 29.5%, *p* = 0.64) were more frequent overall than in our cohort (14.4 vs. 18.1; *p* = 0.005), but as in ours they were balanced between M1 and M2 occlusions.

Patient selection and decision making for MT in isolated M2 occlusions is challenging, as current guidelines are rather vague (class IIb) [2]. In contrast from M1 occlusions, where the general current approach appears to be IVT and MT, a patient’s eligibility for intravenous thrombolysis seems to play a greater role in MT decision-making for M2 occlusions [32]. In a survey by Kappelhof et al., even in IVT patients more than half of the physicians stated that they would perform MT without waiting for the alteplase effect [32] and many (59%) neurointerventionalists would even immediately proceed to MT without prior IVT [33]. Treatment decision-making is further complicated by the diversity of clinical symptoms patients can present with, which are dependent on the eloquence of the affected area [31]. For example, a patient with a right-sided small branch anterior M2 occlusion may barely suffer from any deficits, but a similarly sized left-sided M2 occlusion may result in severe aphasia [32]. The overall necessity to perform MT in M2 occlusions was illustrated in an analysis by Lima et al. [3], where patients with a baseline NIHSS score ≥ 10 but without recanalization therapy achieved good functional outcome in only 22.7% of cases (mRS 0–2) at 90 days, while 40.9% died. Conversely, Sarraj et al. [34] found significantly improved outcomes with MT in patients with M2 occlusions, when compared to best medical treatment. Similar results were also obtained by IAT in a subgroup analysis of PROACT-II (Prolyse in Acute Cerebral Thromboembolism II) [4], with good clinical outcome achieved in only 28.6% of patients after best medical management, but in 48.6% of patients after MT. The impact of successful reperfusion has been shown in the ETIS Registry (excellent outcome (OR 2.3, 95% CI 0.98 to 5.36; *p* = 0.053), favorable outcome (OR 2.79, 95% CI 1.31 to 5.93; *p* = 0.007), and reduced 90-day mortality (OR 0.39, 95% CI 0.19 to 0.79; *p* < 0.01) [24], while Boyanpally et al. found no additional benefit of MT compared to best medical management of M2 lesions [35].

In our cohort, the median baseline NIHSS was 11.00 [IQR 6.25–16.00], and 46.5% had a good functional outcome with a mortality rate of 22.9% (compared to 22.7% and 40.9% in the untreated collective by Lima et al.). This does strongly support the impact of MT when compared to the untreated natural course. However, we cannot prove this within our own dataset due to a missing untreated control group.

New generation stent retrievers dedicated to treating small vessel occlusions with either reduced radial force or smaller crossing profile (or both) are now available to neurointerventionalists. They typically require a smaller microcatheter lumen, thus potentially making catheterization of small caliber and tortuous vessel segments more feasible and safer. However, data on the safety and efficacy of thrombectomy in small vessels with these dedicated devices is limited. In our series, dedicated svSR were used in 17.4% of M2 occlusions and only in 3% of M1 lesions. This approach did not however result in a higher rate of successful reperfusion, a difference in the number of passages or first pass mTICI 2/3, and most importantly, it did not affect the rate of adverse events when compared to M1 segment occlusions. While first pass success was rather low in all subgroups, CA resulted in a higher rate of first pass mTICI 2b/3 in M2 occlusions. With the overall numbers being small, we cannot enforce CA as a first-line approach based on this experience, especially since the ASTER trial [36] found the CA group to have a higher 90-day mortality rate when compared to the stent retriever group (19.6% versus 3.3%; *p* = 0.078). Concerning revascularization success of M2-MT in terms of TICI 3 only, the use of a conventional cSR resulted in the highest rates of mTICI 3 but without impact on clinical improvement in our subgroup-analysis. In the meta-analysis of Saber et al. [23], recanalization rates were 81% (95% CI 79% to 84%), and were equally comparable for cSR versus CA (OR 1.05; 95% CI 0.91 to 1.21). In the post hoc analysis of the ASTER trial [36], these numbers were in the same range (83.9% vs. 89.6%; *p* = 0.36), both without any significant differences in terms of clinical success. 

Currently, efforts to create more evidence on M2-MT are underway. The ongoing REVISAR trial (ClinicalTrials.gov Identifier: NCT04479020) with the APERIO^®^ or APERIO^®^ Hybrid may clarify the peri-interventional risk profile and recanalization rates with svSR. The results will be helpful until we finally have data from randomized trials, such as the ESCAPE-MeVO (https://aspectsinstroke.com/mevo1/inclusion-criteria-2/escape-mevo-info (accessde on 25 June, 2022)), DISTAL (DISTAL | Department of Clinical Research (unibas.ch)) and DISCOUNT (ClinicalTrials.gov Identifier: NCT05030142) which will hopefully provide more robust clinical evidence. 

The observational study design without controlled and randomized data and the absence of a control group did not allow us to draw conclusions about causality concerning treatment effects. We cannot rule out a selection bias induced by the multicenter design with different participating centers and with respect to their treatment standards regarding IVT or acute stenting, but both groups were treated by the same teams of neurologists and neurointerventionalists, likely diminishing these effects. 

## 5. Conclusions

In the current absence of randomized controlled trials evaluating MT in M2 occlusions, neurologists and neurointerventionalists are challenged to make decisions if and how to approach isolated M2 occlusions by MT in everyday practice. While the safety and efficacy of MT are well proven for LVO including M1 lesions, supporting evidence is rather scarce for M2 occlusions. When performing MT in M2 occlusions, we thus need to carefully balance treatment safety and efficacy as was the case for LVO treatment prior to the major trials published in 2015. Our results are in keeping with previous work, suggesting that M2 MT is equally feasible, safe, and clinically effective as it is in M1 occlusions. The use of dedicated svSR did not affect the safety nor the efficacy of the treatment in our cohort. The upcoming randomized trials are thus necessary to gather the missing evidence concerning indication and choice of the most appropriate therapeutic approach.

## Figures and Tables

**Table 1:** Baseline characteristics MCA-M1 vs. MCA-M2.

	MCA-M1 (n = 2689)	MCA-M2 (n = 1115)	*p* Value
**Age—years, median (IQR)**	76.00 [65.00–82.00]	77.00 [66.00–83.00]	0.41
**Sex—female, n (%)**	1449 (53.9)	550 (49.5)	0.14
**Clinical characteristics at admission**			
**Baseline NIHSS at ADM, median (IQR)**	15.00 [10.00–18.00]	11.00 [6.25–16.00]	**<0.001**
**Time intervals (minutes)**			
**Symptom onset to groin**	195.00 [140.00–265.50]	190.00 [139.00–270.00]	0.99
**Last seen well to groin**	490.00 [301.00–814.50]	520.50 [307.25–811.75]	0.89
**IVT treatment, n (%)**	1335 (50.0)	609 (54.8)	0.80
**ASPECTS, median (IQR)**	9.00 [7.00–10.00]	9.00 [8.00–10.00]s	**<0.001**
**Type of anesthesia**			0.15
**Conscious sedation, n (%)**	792 (30.9)	283 (26.2)	
**General anesthesia, n (%)**	1685 (65.7)	762 (70.6)	
**Conversion, n (%)**	89 (3.5)	35 (3.2)	

Abbreviations: ADM: Admission; ASPECTS: Alberta Stroke Program Early CT Score; ICA: intracranial carotid artery. ICH: intracerebral hemorrhage. IVT: intravenous thrombolysis. IQR: interquartile range. MCA: middle cerebral artery. mRS: modified Rankin Scale. NIHSS: National Institute of Health Stroke Scale. Values in bold indicate significant differences at the 5% level of significance.

**Table 2:** Outcomes at 90 days, procedural results, and safety outcome.

	MCA-M1 (n = 2689)	MCA-M2 (n = 1115)	*p* Value
** Outcome **			
**Good functional outcome (mRS 0–2), n (%)**	1348 (38.9)	607 (46.5)	**<0.001**
**Mortality (mRS 6), n (%)**	590 (26.9)	218 (22.9)	0.23
**Improvement of clinical symptomatic, n (%)**	1359 (62.5)	600 (61.4)	0.57
**mTICI, n (%)**			0.30
**0**	183 (6.9)	90 (8.2)	
**1**	28 (1.0)	20 (1.8)	
**2a**	136 (5.1)	75 (6.8)	
**2b**	889 (33.3)	393 (35.6)	
**3**	1431 (53.7)	525 (47.6)	
** Procedural results **			**<0.001**
**Aspiration catheter only, n (%)**	401 (14.9)	124 (11.1)	
**Conventional stent retriever ± aspiration, n (%)**	2062 (76.7)	710 (63.7)	
**Small vessel stent retriever ± aspiration, n (%)**	80 (3.0)	194 (17.4)	
**Small vessel+ conventional stent retriever ± aspiration, n = (%)**	39 (1.5)	15 (1.3)	
**Number of passages, median (IQR)**	2.00 [2.00–8.00]	2.00 [2.00–7.00]	0.10
**First pass mTICI 2b/3**	34 (1.3)	29 (2.7)	0.50
**Treatment completed, n (%)**	2505 (94.2)	1010 (91.6)	0.40
**Treatment extracranial ICA (NASCET > 70%), n (%)**	326 (12.1)	96 (8.6)	0.20
**Treatment extracranial ICA with stent, n (%)**	237 (8.8)	74 (6.6)	0.30
** Safety outcome **			
**Periprocedural complications, n (%)**	385 (14.4)	202 (18.1)	0.63
**Allergic**	4 (0.1)	2 (0.2)	
**Reanimation**	6 (0.2)	1 (0.1)	
**Periprocedural rethrombosis**	12 (0.4)	4 (0.4)	
**SAH, n (%)**	43 (1.6)	29 (2.6)	0.53
**Vasospasm, n (%)**	96 (3.6)	49 (4.4)	0.26
**Recurrent stroke 24, n (%)**	91 (3.4)	44 (3.9)	0.44
**ICH 24 h, n (%)**	298 (11.1)	124 (11.1)	1.0
**Hemorrhagic transformation 24 h, n (%)**	91 (3.4)	44 (3.9)	0.45

Abbreviations: ADM: Admission; DC: discharge; ICA: intracranial carotid artery. ICH: intracerebral hemorrhage. IQR: interquartile range. MCA: middle cerebral artery. mRS: modified Rankin Scale. mTICI: modified thrombolysis in cerebral infarction score. Values in bold indicate significant differences at the 5% level of significance.

**Table 3:** Baseline characteristics sub-analysis MCA-M2 occlusions.

	Aspiration Only (CA) (n = 124)	Conventional Stent Retriever ± Aspiration (n = 710)	Small Vessel Stent Retriever ± Aspiration (n = 194)	*p* Value
Age—years, median (IQR)	76.00 [63.75–84.00]	77.00 [67.00–83.00]	77.00 [68.00–82.00]	0.75
Sex—female, n (%)	54 (43.5)	361 (51.1)	95 (49.0)	0.29
Clinical characteristics at admission				
Baseline NIHSS at ADM, median (IQR)	11.00 [7.00–15.25]	11.00 [6.00–16.00]	10.00 [6.00–16.00]	0.83
Time intervals (minutes)				
Symptom onset to groin	159.00 [135.00–217.25]	195.00 [139.50–276.50]	186.50 [134.00–267.00]	0.14
Last seen well to groin	446.00 [370.00–761.50]	538.50 [304.75–837.00]	525.00 [275.00–853.50]	0.97
IVT treatment, n (%)	80 (64.5)	361 (51.0)	114 (59.1)	0.60
ASPECTS, median (IQR)	9.00 [8.00–10.00]	9.00 [8.00–10.00]	9.00 [8.00–10.00]	0.34

Abbreviations: ADM: Admission; ASPECTS: Alberta Stroke Program Early CT Score; IVT: intravenous thrombolysis. IQR: interquartile range. MCA: middle cerebral artery. mRS: modified Rankin Scale. NIHSS: National Institute of Health Stroke ScalemTICI: modified thrombolysis in cerebral infarction score.

**Table 4:** Sub-analysis MCA-M2 occlusions, outcomes at 90 days, procedural results, and safety outcome.

	Aspiration Only (CA) (n = 124)	Conventional Stent Retriever ± Aspiration (n = 710)	Small Vessel Stent Retriever ± Aspiration (n = 194)	*p* Value
** Outcome **				
**Good functional outcome (mRS 0-2), n (%)**	71 (50.5)	385 (45.7)	115 (51.8)	0.65
**Mortality (mRS 6), n (%)**	23 (21.5)	140 (23.4)	34 (20.7)	0.74
**Improvement of clinical symptomatic, n (%)**	75 (71.4)	369 (60.5)	110 (60.8)	0.98
**mTICI, n (%)**				
**0**	12 (9.8)	31 (4.4)	22 (11.4)	1.0
**1**	5 (4.1)	8 (1.1)	3 (1.6)	0.52
**2a**	10 (8.2)	44 (6.2)	10 (5.2)	0.56
**2b**	49 (40.2)	241 (34.2)	77 (39.9)	0.21
**3**	46 (37.7)	380 (54.0)	81 (42.0)	**<0.001**
** Procedural results **				
**Number of passages, median (IQR)**	2.00 [2.00–2.00]	7.00 [2.00–8.00]	2.00 [2.00. 7.00]	**<0.001**
**First pass mTICI 2b/3**	5 (4.2)	1 (0.1)	0 (0.0)	**<0.001**
**Treatment completed, n (%)**	116 (93.5)	683 (97.2)	182 (95.3)	0.96
**Treatment extracranial ICA (NASCET > 70%), n (%)**	9 (7.3)	61 (8.6)	16 (8.2)	0.88
**Treatment extracranial ICA with stent, n (%)**	8 (6.5)	52 (7.3)	10 (5.2)	0.56
** Safety outcome **				
**Periprocedural complications, n (%)**	18 (14.5)	128 (18.1)	42 (21.6)	0.20
**SAH, n (%)**	1 (0.8)	20 (2.8)	8 (4.1)	0.22
**Vasospasm, n (%)**	4 (3.2)	39 (5.5)	5 (2.6)	0.17
**Recurrent stroke 24 h, n (%)**	1 (0.8)	3 (0.4)	0 (0.0)	0.62
**ICH 24 h, n (%)**	9 (7.3)	79 (11.1)	22 (11.3)	0.42
**Hemorrhagic transformation 24 h, n (%)**	2 (1.6)	10 (1.4)	1 (0.5)	0.57

Abbreviations: ADM: Admission; DC: discharge; ICA: intracranial carotid artery. ICH: intracerebral hemorrhage. IQR: interquartile range. MCA: middle cerebral artery. mRS: modified Rankin Scale mTICI: modified thrombolysis in cerebral infarction score.

## Data Availability

The data that support the findings of this study are available from the corresponding author on reasonable request.

## Supplementary Materials

The following supporting information can be downloaded at: https://www.mdpi.com/article/10.3390/jcm11154619/s1, Table S1: Missing individual variables. Table S2: Missing individual treatment variables.

## Appendix A

**Table A1:** GSR-Investigators.

Name	Degree	Organization	Role	Contribution
Tobias Boeckh-Behrens	MD	Klinikum r.d.Isar, Munich, Germany	Site Investigator	German Stroke Registry—Steering Committee
Silke Wunderlich	MD	Klinikum r.d.Isar, Munich, Germany	Site Investigator	German Stroke Registry—Steering Committee
Arno Reich	MD	Uniklinik RWTH Aachen, Germany	Site Investigator	German Stroke Registry—Steering Committee
Anastasios Mpotsaris	MD	Uniklinik RWTH Aachen, Germany	Site Investigator	German Stroke Registry—Steering Committee
Martin Wiesmann	MD	Uniklinik RWTH Aachen, Germany	Site Investigator	German Stroke Registry—Steering Committee
Ulrike Ernemann	MD	Tübingen University Hospital, Germany	Site Investigator	German Stroke Registry—Steering Committee
Till-Karsten Hauser	MD	Tübingen University Hospital, Germany	Site Investigator	German Stroke Registry—Steering Committee
Christian H Nolte	MD	Charite Campus Benjamin Franklin	Site Investigator	German Stroke Registry—Steering Committee
Eberhard Siebert	MD	Charité – Campus Benjamin Franklin und Campus Charité Mitte, Berlin, Germany	Site Investigator	German Stroke Registry—Steering Committee
Sarah Zweynert	MD	Charité—Campus Virchow Klinikum, Berlin, Germany	Site Investigator	German Stroke Registry—Steering Committee
Georg Bohner	MD	Charité—Campus Virchow Klinikum, Berlin, Germany	Site Investigator	German Stroke Registry—Steering Committee
Alexander Ludolph	MD	Sana Klinikum Offenbach, Germany	Site Investigator	German Stroke Registry—Steering Committee
Karl-Heinz Henn	MD	Sana Klinikum Offenbach, Germany	Site Investigator	German Stroke Registry—Steering Committee
Waltraud Pfeilschifter	MD	Uniklinik Frankfurt/ Main, Germany	Site Investigator	German Stroke Registry—Steering Committee
Marlis Wagner	MD	Uniklinik Frankfurt/ Main, Germany	Site Investigator	German Stroke Registry—Steering Committee
Joachim Röther	MD	Asklepios Klinik Altona, Hamburg, Germany	Site Investigator	German Stroke Registry—Steering Committee
Bernd Eckert	MD	Asklepios Klinik Altona, Hamburg, Germany	Site Investigator	German Stroke Registry—Steering Committee
Jörg Berrouschot	MD	Klinikum Altenburger Land, Altenburg, Germany	Site Investigator	German Stroke Registry—Steering Committee
Albrecht Bormann	MD	Klinikum Altenburger Land, Altenburg, Germany	Site Investigator	German Stroke Registry—Steering Committee
Anna Alegiani	MD	University Medical Center Hamburg-Eppendorf, Hamburg, Germany	Site Investigator	German Stroke Registry—Steering Committee
Jens Fiehler	MD	University Medical Center Hamburg-Eppendorf, Hamburg, Germany	Site Investigator	German Stroke Registry—Steering Committee
Fabian Flottmann	MD	University Medical Center Hamburg-Eppendorf, Hamburg, Germany	Site Investigator	German Stroke Registry—Steering Committee
Christian Gerloff	MD	University Medical Center Hamburg-Eppendorf, Hamburg, Germany	Site Investigator	German Stroke Registry—Steering Committee
Götz Thomalla	MD	University Medical Center Hamburg-Eppendorf, Hamburg, Germany	Site Investigator	German Stroke Registry—Steering Committee
Elke Hattingen	MD	University Hospital Bonn, Germany	Site Investigator	German Stroke Registry—Steering Committee
Gabor Petzold	MD	University Hospital Bonn, Germany	Site Investigator	German Stroke Registry—Steering Committee
Sven Thonke	MD	Klinikum Hanau, Germany	Site Investigator	German Stroke Registry—Steering Committee
Christopher Bangard	MD	Klinikum Hanau, Germany	Site Investigator	German Stroke Registry—Steering Committee
Christoffer Kraemer	MD	Klinikum Lüneburg, Germany	Site Investigator	German Stroke Registry—Steering Committee
Martin Dichgans	MD	Ludwig Maximilian University of Munich, Germany	Site Investigator	German Stroke Registry—Steering Committee
Marios Psychogios	MD	Georg-August-Universität Göttingen, Germany	Site Investigator	German Stroke Registry—Steering Committee
Jan Liman	MD	Georg-August-Universität Göttingen, Germany	Site Investigator	German Stroke Registry—Steering Committee
Martina Petersen	MD	Klinikum Osnabrück, Germany	Site Investigator	German Stroke Registry—Steering Committee
Florian Stögbauer	MD	Klinikum Osnabrück, Germany	Site Investigator	German Stroke Registry—Steering Committee
Peter Kraft	MD	University Hospital Würzburg, Germany	Site Investigator	German Stroke Registry—Steering Committee
Mirko Pham	MD	University Hospital Würzburg, Germany	Site Investigator	German Stroke Registry—Steering Committee
Michael Braun	MD	Bezirkskrankenhaus Günzburg, Germany	Site Investigator	German Stroke Registry—Steering Committee
Gerhard F. Hamann	MD	Bezirkskrankenhaus Günzburg, Germany	Site Investigator	German Stroke Registry—Steering Committee
Andreas Kastrup	MD	Klinikum Bremen Mitte, Germany	Site Investigator	German Stroke Registry—Steering Committee
Christian Roth	MD	Klinikum Bremen Mitte, Germany	Site Investigator	German Stroke Registry—Steering Committee
Klaus Gröschel	MD	University Medical Center Mainz, Germany	Site Investigator	German Stroke Registry—Steering Committee
Timo Uphaus	MD	University Medical Center Mainz, Germany	Site Investigator	German Stroke Registry—Steering Committee
Volker Limmroth	MD	Kliniken Köln, Germany	Site Investigator	German Stroke Registry—Steering Committee
