# Supplementary material for: Middle Cerebral Artery M2 Thrombectomy: Safety and Technical Considerations in the German Stroke Registry (GSR)
Source: J Clin Med. 2022 Aug 8;11(15):4619. doi: 10.3390/jcm11154619 (PMC9369518; doi:10.3390/jcm11154619)
Supplement: Supplementary file 1 [file jcm-11-04619-s001.zip › jcm-1812686-supplementary.pdf]

Online Supplement:

Table S1: Missing individual variables.

	MCA-M1 (n = 2689)	MCA-M2 (n= 1115)
Age	1	3
Sex	0	3
NIHSS_adm	196	33
NIHSS_DC	506	138
mRS_DC	214	50
mRS	493	165
ASPECTS	291	94
Treat_aci_70	0	0
Treat_extracranial_stent	0	0
Treat_ivt	17	3
Treatment_completed	29	12
Anesthesia	123	35
Treatment_device	1	1
N_pass	146	66
TICI	22	12
Treat_ae	22	2
Treat_ae_ICH	0	0
Treat_ae_vasospasm	0	0
Treat_ae_other	0	0
f24h_ae_ICH	0	0
f24h_ae_recStroke	0	0
Diff_SO_GRO	1226	430
Diff_LSW_GRO	1859	807
SAB_Treat_ae	0	0
SAB_f24h	0	0
haem_trafo_f24h	0	0
Segment	0	0

**mRS_c**	493	165
**death**	493	165
**NIHSS_improvement**	516	138
**first_pass**	116	48

Table S2: Missing individual variables.

	**Aspiration only (CA)** (n = 124)	**Conventional stent retriever ± aspiration** (n= 710)	**Small vessel stent retriever ± aspiration** (n= 194)
**Age**	1	3	2
**Sex**	1	4	1
**NIHSS_adm**	5	28	3
**NIHSS_DC**	20	101	14
**mRS_DC**	5	42	6
**mRS**	18	112	31
**ASPECTS**	11	62	19
**Treat_aci_70**	1	1	1
**Treat_extracranial_stent**	1	1	1
**Treat_ivt**	1	3	2
**Treatment_completed**	1	8	4
**Anesthesia**	4	25	8
**Treatment_device**	1	1	1
**N_pass**	14	33	9
**TICI**	3	7	2
**Treat_ae**	1	3	1
**Treat_ae_ICH**	1	1	1
**Treat_ae_vasospasm**	1	1	1
**Treat_ae_other**	1	1	1
**f24h_ae_ICH**	1	1	1
**f24h_ae_recStroke**	1	1	1
**Diff_SO_GRO**	53	272	71
**Diff_LSW_GRO**	86	519	145

**SAB_Treat_ae**	1	1	1
**SAB_f24h**	1	1	1
**haem_trafo_f24h**	1	1	1
**Segment**	1	1	1
**mRS_c**	18	112	31
**death**	18	112	31
**NIHSS_improvement**	20	101	14
**first_pass**	7	27	4
